# Rotavirus Antigenemia in Children Is Associated with Viremia

**DOI:** 10.1371/journal.pmed.0040121

**Published:** 2007-04-17

**Authors:** Sarah E Blutt, David O Matson, Sue E Crawford, Mary Allen Staat, Parvin Azimi, Berkeley L Bennett, Pedro A Piedra, Margaret E Conner

**Affiliations:** 1 Department of Molecular Virology and Microbiology, Baylor College of Medicine, Houston, Texas, United States of America; 2 Michael E. Debakey Veterans Affairs Medical Center, Houston, Texas, United States of America; 3 Center for Pediatric Research, Norfolk, Virginia, United States of America; 4 Cincinnati Children's Hospital Medical Center, Cincinnati, Ohio, United States of America; 5 Children's Hospital of Oakland, Oakland, California, United States of America; Royal West Sussex NHS Trust, United Kingdom

## Abstract

**Background:**

Antigenemia is commonly detected in rotavirus-infected children. Although rotavirus RNA has been detected in serum, definitive proof of rotavirus viremia has not been shown. We aimed to analyze a defined patient population to determine if infectious virus could be detected in sera from children with rotavirus antigenemia.

**Methods and Findings:**

Serum samples obtained upon hospitalization from children with gastroenteritis (57 stool rotavirus-positive and 41 rotavirus-negative), children with diagnosed bronchiolitis of known (*n* = 58) or unknown (*n* = 17) viral etiology, children with noninfectious, nonchronic conditions (*n* = 17), and healthy adults (*n* = 28) were tested for rotavirus antigen by enzyme immunoassay (EIA). Results of serum antigen testing were assessed for association with clinical and immunological attributes of the children. Rotavirus antigenemia was detected in 90% (51/57) of children with rotavirus-positive stools, in 89% (8/9) of children without diarrhea but with rotavirus-positive stools, in 12% (2/17) of children with bronchiolitis of unknown etiology without gastroenteritis, and in 12% (5/41) of children with gastroenteritis but with rotavirus-negative stools. Antigenemia was not detected in sera from children with noninfectious nonchronic conditions, children with bronchiolitis of known etiology and no gastroenteritis, or healthy adults. Neither age nor timing of serum collection within eight days after onset of gastroenteritis significantly affected levels of antigenemia, and there was no correlation between antigenemia and viral genotype. However, there was a negative correlation between serum rotavirus antigen and acute rotavirus-specific serum IgA (*r* = −0.44, *p* = 0.025) and IgG (*r* = −0.40, *p* = 0.01) titers. We examined 11 antigen-positive and nine antigen-negative sera for infectious virus after three blind serial passages in HT-29 cells using immunofluorescence staining for rotavirus structural and nonstructural proteins. Infectious virus was detected in 11/11 (100%) sera from serum antigen-positive children and in two out of nine (22%) sera samples from antigen-negative children (*p* = 0.002).

**Conclusions:**

Most children infected with rotavirus are viremic. The presence of viremia is directly related to the detection of antigenemia and is independent of the presence of diarrhea. Antigenemia load is inversely related to the titer of antirotavirus antibody in the serum. The finding of infectious rotavirus in the blood suggests extraintestinal involvement in rotavirus pathogenesis; however, the impact of rotavirus viremia on clinical manifestations of infection is unknown.

## Abstract 2

A study of 57 children with rotavirus-positive stools found that most were viremic, and that the presence of viremia was directly related to antigenemia and independent of the presence of diarrhea.

## Editors' Summary

**Background.:**

Rotavirus is a type of virus that is the commonest cause of severe diarrhea among children worldwide. It is passed from one person to another when virus present in the stool of an infected person is swallowed by another individual. The infection causes vomiting, watery diarrhea, and fever; many children need to be hospitalized as a result and globally more than 600,000 children are thought to die as a result of rotavirus infections per year. Evidence from single case descriptions of infected children have suggested that rotavirus might also cause symptoms outside of the gut—for example, in the lungs or brain. Previous studies have found fragments of rotavirus, for example RNA or parts of virus protein, in tissues outside of the gut such as liver, kidney, blood, and heart. However, simply finding fragments such as RNA or protein does not necessarily mean that rotavirus infects these tissues.

**Why Was This Study Done?:**

These researchers wanted to find out whether rotavirus was present in the blood of infected children. If evidence of rotavirus in the blood was found, this might help explain why some children infected with rotavirus have symptoms affecting organs other than the gut.

**What Did the Researchers Do and Find?:**

In this study, five groups of patients were recruited and tests were done on each to find out whether infectious rotavirus was present in their bloodstream, and also whether the researchers could detect rotavirus components in blood using antibodies against particular parts of the rotavirus particle. The five groups of patients that were compared included children hospitalized with gastroenteritis; children hospitalized with noninfectious conditions; healthy adult laboratory workers; children with lung infections from known viruses; and finally children with lung infections of unknown cause. The researchers found that among the children with gastroenteritis who had rotavirus in their stool, 90% also had evidence of rotavirus particles in their bloodstream. By contrast, control individuals (either children who were hospitalized with noninfectious conditions or healthy adults) did not have rotavirus particles in blood. A small proportion of children with gastroenteritis but no rotavirus in their stool did have rotavirus particles in blood. Interestingly, a small proportion of the children who had lung infections (but in whom no known virus had been identified as the cause) showed evidence of rotavirus in their bloodstreams. Finally, in a group of 11 children with evidence of rotavirus particles in their bloodstreams, all were found to also have infectious virus present in the blood.

**What Do These Findings Mean?:**

These results show that rotavirus is able to spread beyond the gut and into the bloodstream. The finding that rotavirus can spread into the bloodstream may explain some earlier suggestions that rotavirus is responsible for symptoms outside of the gut. However, it is not yet clear how commonly children with rotavirus have other symptoms resulting from the virus spreading into their bloodstream.

**Additional Information.:**

Please access these Web sites via the online version of this summary at http://dx.doi.org/10.1371/journal.pmed.0040121.

- Read the related *PLoS Medicine* Perspective article by David Candy
- Information from the World Health Organization Initiative for Vaccine Research on rotavirus disease burden; see also the Rotavirus Vaccine Program, a partnership that aims to develop rotavirus vaccines appropriate for use in developing countries
- Information from the US Centers for Disease Control and Prevention about rotavirus
- Health Encyclopedia entry from the UK's NHS Direct on Rotavirus

## Introduction

Rotavirus is a major cause of gastroenteritis in pediatric populations, resulting in 114 million episodes of gastroenteritis per year worldwide [1] and an annual economic burden in the United States estimated in 1998 at over one billion dollars [2]. Rotaviral infection is generally thought to be localized to the epithelial cells lining the small intestine. However, case reports suggest that rotavirus may cause infection and illness outside the intestine, including hepatitis and nephritis [3], pneumonia [4], exanthema [5], disseminated intravascular coagulation [6], haemophagocytic lymphohistiocytosis [7], and neurological complications such as encephalitis [8], encephalopathy [7], cerebellitis [9], or convulsions or seizures [10,11]. Reports of attempts to find rotavirus RNA or proteins at extraintestinal sites include detection of RNA in cerebral spinal fluid [12,13], central nervous system [14], heart [14,15], blood [16], and endothelial cells [14,17], while rotavirus nonstructural proteins were detected in liver and kidney sections [3]. These findings support the possibility of uncommon extraintestinal infections that were thought to be either infections with unusual rotavirus strains or rare host genetic or immunologic defects in the infected child. Early work supporting this idea described rotavirus antigenemia in immunodeficient but not immune-competent children [18].

Contrary to the general assumption that rotavirus remains confined to the intestine during infection, rotavirus antigenemia was reported in 67% of immunocompetent children with rotavirus diarrhea [19], 43% of sera from Jamaican children during a gastroenteritis outbreak [20], and acute phase sera from five of eight children experiencing central nervous system complications [21] with rotavirus gastroenteritis. The ability to detect rotavirus antigenemia is influenced by factors such as increased acute-phase serum IgG antibody titer [20], days after onset of diarrhea [19,20], and stool viral antigen levels [20]. Although these studies indicate that rotavirus antigenemia is common, they used small or undefined patient and control populations and archived specimens. More comprehensive studies with well-defined patient populations are necessary to better understand the host and viral factors that influence the extraintestinal spread of rotavirus. Here, we report examination of samples from a prospective case-surveillance study and a prospective cohort study to define some of the clinical and immunological parameters that influence rotavirus antigenemia.

Rotavirus RNA is present in both extraintestinal tissues and blood from children with rotavirus diarrhea [16,19,20,22]. Detection rates of extraintestinal RNA in blood have varied widely, ranging from 0%–64% in rotavirus-infected children and are not always concordant with presence of antigenemia detected by enzyme immunoassay (EIA) [20,23,24]. Furthermore, the presence of RNA may not always be indicative of infectious virus. Proof that rotavirus antigenemia or the detection of RNA in the sera reflects infectious virus in the sera (viremia) of infected children has not been reported. Viremia has been detected in rotavirus-infected animals, including mice, rats, and piglets [25]. Direct cultivation of primary stool isolates of human rotaviruses in animals or tissue culture cells is not routinely successful, and cultivation from serum poses additional challenges. We utilized an amplification approach and adaptation in cell culture to investigate whether rotavirus antigen-positive serum samples from children contain infectious rotavirus.

## Methods

### Enrollment, Sample Collection, and Informed Consent

We examined antigenemia in five patient groups in three separate studies. Study 1 included children from active surveillance efforts to identify rotavirus-positive and -negative children, and hospitalized matched controls with noninfectious nonchronic conditions. Study 2 included healthy adult laboratory workers. Study 3 included children with bronchiolitis in which a viral agent could not be isolated from a nasal wash specimen, and as a control group, children with virus-associated bronchiolitis determined by isolation of a virus from nasal washes by tissue culture.

#### Study 1: Children from active surveillance to identify rotavirus positive and negative children.

Serum and stool samples collected from November 1997 through December 1999 during an active, prospective case-surveillance study at three free-standing pediatric hospitals in the United States [26,27] were analyzed. Criteria for eligibility in this study were acute illness of less than 7 d duration characterized by diarrhea, vomiting without a respiratory or structural gastrointestinal tract cause, and/or fever of unknown cause. Fever was a criterion for inclusion in the active surveillance study but not a criterion for inclusion in this study. Diarrhea was defined as an episode of three or more stools in a 24-h period judged by the caregiver to be looser than normal. Vomiting was defined as the forceful expulsion of gastric contents occurring at least once in a 24-h period. Fever was defined as a rectal temperature of >38.0 °C. After informed consent was obtained, the parent or caregiver was interviewed to obtain information regarding the symptoms of the child's illness, medical history, and demographic information.

As a comparison group for children in study 1 hospitalized with gastroenteritis, a set of serum samples was obtained from an age-matched population that included children hospitalized for noninfectious, nonchronic conditions. These samples were collected during the same time period and at the same sites as samples collected from children with gastroenteritis.

#### Study 2: Healthy adult laboratory workers.

Sera from adult laboratory workers without gastroenteritis were obtained.

#### Study 3: Children with bronchiolitis.

Sera collected during a prospective cohort study of children less than 24 mo of age who presented to the emergency department between November 2004 and February 2005 at Texas Children's Hospital, Houston, Texas, United States with signs and symptoms of bronchiolitis (lower airway disease with tachypnea, crackles, wheezing, or retractions), combined with a history of preceding upper airway infection (nasal congestion or rhinorrhea), were also examined. Sera collection overlapped with rotavirus season in Houston. Nasal washes collected from these children were analyzed for the presence of respiratory viruses, which included influenza A or B, human metapneumovirus, respiratory syncytial virus, parainfluenza viruses, picornoviruses, and adenoviruses as described (B. L. Bennett, S. G. Cron, R. L. Atmar, P. A. Piedra unpublished data).

Informed consent was obtained from parents or guardians of children enrolled in the study. The Institutional Review Boards at each site approved each study.

### Sample Analysis

Stool (active, prospective case surveillance) and serum (active, prospective case surveillance and bronchiolitis study) specimens were collected at enrollment (within 24 h of admission) and stored at 4 °C and −70 °C, respectively, until testing. Stool specimens were tested for rotavirus antigen using a commercial EIA (Rotaclone, Meridian Bioscience, http://www.meridianbioscience.com). If the study participant's stool tested rotavirus-negative and an acute serum had been obtained, a convalescent blood sample was solicited. The concentration of rotaviral antigen in sera was estimated by EIA as previously described, with the following modifications [28]: 96-well polyvinylchloride plates were coated with 50 μl of a mouse monoclonal anti-VP6 antibody (6E7, raised against rotavirus strain SA11) overnight at room temperature. 6E7 is broadly cross-reactive, recognizing many different strains of animal and human rotavirus, including EC_wt_ (P[19]G3, murine), EDIM (P[18]G3, murine), RRV (P[3]G3, rhesus), SA11 (P[2]G3, simian), ALA (P[14]G3, lapine), HAL1166 (P[14]G8, human), Wa (P[8]G1, human), PA169 (P[14]G6, human), Ito (P[8]G3, human), and Hochi (P[8]G4, human) (unpublished data). Wells were blocked with 200 μl of 5% Carnation instant milk in PBS (blotto) for 2 h at 37 °C. Serum samples were diluted 1:10 in TNC (10 mM Tris [pH 7.4], 140 mM NaCl, 10 mM CaCl_2_) to a total volume of 50 μl. Samples were treated for 10 min at room temperature with 25 mM EDTA. Hyperimmune guinea pig antiserum to rotavirus (strain ALA) diluted in 0.5% blotto was used as a detector antibody followed by HRP-conjugated goat anti-guinea pig IgG (Sigma-Aldrich, http://www.sigmaaldrich.com) diluted in 0.5% blotto with 2.5% fetal calf serum. Antigenemia was expressed as the EIA (OD)_450 nm_ value of each sample × 1,000. The mean OD value plus two standard deviations was calculated from OD values obtained from the sera of children with noninfectious, nonchronic conditions, and healthy adults and used as a cut-off value. A sample with an OD above the cut-off value was considered positive. All samples were coded, analyzed by EIA, and decoded after testing. Sera were analyzed for antirotavirus IgA and IgG antibody using a standardized YO (P[8]G3) strain human rotavirus as antigen as described earlier [29,30]. An antibody response was defined as a 4-fold rise in antibody titer and/or a conversion from negative to positive in an acute/convalescent serum pair. Validation of serum antibody testing was established by utilization of the same antigen preparation and by interspersion of 30 sera of known titer tested previously in establishment of an immune correlate for protection against rotavirus infection [29,30]. P- and G-typing was performed as described [31–33].

### Determination of Viremia

Infectious virus from human serum was propagated in HT-29 cells using a modification of the method in Superti et al. [34]. To amplify potentially infectious virus, serum samples from the active, prospective case-surveillance study were serially passaged three to five times in HT-29 cells as previously described [35]. Serial 2-fold dilutions of serum from 1:20 to 1:160 or 1:20 to 1:2560 were tested in each passage. The presence of infectious virus from the passaged samples was assessed by fluorescent focus assay and detected with either a polyclonal antibody generated against whole virus (rabbit anti-ALA) or the nonstructural protein NSP4 114–135 peptide antibody [36] as previously described [37]. Both antibodies are broadly reactive and recognize multiple animal and human strains of rotavirus ([38] and unpublished data). Fluorescent cells were imaged using an Olympus IX70 inverted fluorescent microscope (http://www.olympusamerica.com). Cells were scored by an observer blinded to the study design. If positive cells were observed at any dilution, the serum was considered positive for infectious virus. Several approaches were utilized to (1) reduce the likelihood of and (2) provide detection of cross contamination during the multiple blind passages. All passages were performed in laminar flow hood. Blank wells and wells with sera from children with noninfectious, nonchronic conditions were always fluorescent negative despite being interspersed between positive wells containing antigen positive sera. A total of six positive and four negative samples were serially passaged in two independent series of experiments starting with the original sera. The presence or absence of infectious virus in the sera in the replicate experiment was exactly the same as the initial result. The pattern of staining, the integrity of the monolayer, and the growth kinetics over several passages was consistent with a virus adapting to tissue culture and not that of a previously adapted laboratory strain.

### Statistical Analysis

OD values for patients in different groups were compared by ANOVA. When Bartlett's test indicated variances of groups significantly differed, the nonparametric Kruskal-Wallis H test value was taken. Serum OD values were scored as positive or negative as described above. Regression was used to test the positive or negative association of the OD values among positive sera with continuous variables or by logistic regression with binomial variables. *p* < 0.05 was considered significant. Statistical analyses were conducted in Epi Info, version 6.0 [39].

## Results

### Rotavirus Antigenemia Does Not Depend upon Excretion of Rotavirus in the Stool or the Clinical Manifestation of Diarrhea

**Figure 1:**
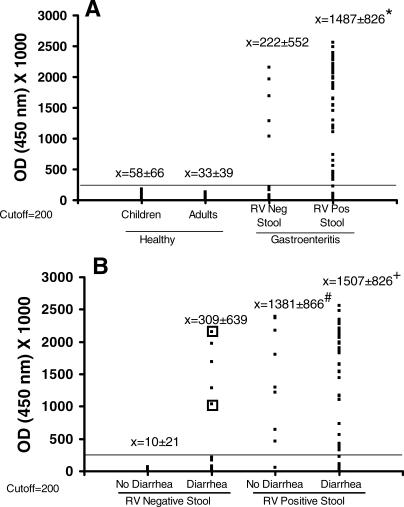
Rotavirus Antigenemia in Children Does Not Depend on the Presence of Diarrhea (A) Sera obtained from children with noninfectious, nonchronic conditions (*n* = 17), healthy adults (*n* = 28), children with gastroenteritis but rotavirus stool negative (*n* = 41), and children with gastroenteritis that were rotavirus stool positive (*n* = 57) were diluted 1:10 and tested for rotavirus antigen by EIA. Values for each sample were recorded as OD × 1,000. The cut-off value for positive samples (line) was established by calculating two standard deviations above the mean OD value of the children with noninfectious, nonchronic conditions and healthy adults. Numbers indicate mean OD values ± standard deviation. *p = 0.001 compared to children with noninfectious, nonchronic conditions. (B) Children with gastroenteritis (*n* = 98) with or without rotavirus present in the stool were further stratified on the basis of criteria of the presence or absence of diarrhea. Numbers indicate mean OD values ± standard deviation. Boxes indicate children from whom serum samples were analyzed for increases in antibody titers between acute and convalescent samples. ^#^ *p* = 0.001 compared to rotavirus (RV) Neg No Diarrhea group. ^+^ *p* = 0.88 compared to rotavirus Pos No Diarrhea group.

The most common clinical manifestation of rotavirus infection is dehydrating diarrhea. Antigenemia occurs in children with rotavirus diarrhea [19–21], but it is unknown whether diarrhea is a necessary correlate for antigenemia. To investigate this question, we obtained sera and stools from 98 children with gastroenteritis. Of the 57 children whose stool was rotavirus-positive, rotavirus antigenemia was detected in 51 (90%) (Figure 1A). Antigenemia was not observed in any serum collected from either set of controls: children with noninfectious, nonchronic conditions (0/17) or healthy adults (0/28). The mean OD value (*p* = 0.001) and the rate of antigenemia (*p* = 0.001) in the rotavirus-positive stool group were both significantly higher than those of the healthy group.

Of the 41 children with gastroenteritis and rotavirus-negative stools, five (12%) had rotavirus antigenemia (Figure 1A). All five with antigenemia also had diarrhea. Although the mean OD (*p* = 0.001) and rate (*p* = 0.001) were significantly lower than that of the rotavirus-positive stool group (Figure 1A), the mean OD (*p* = 0.67) and rate (*p* = 0.17) were not significantly higher than the rate of 0% among children with noninfectious, nonchronic conditions. From these five children whose stools were rotavirus negative but serum positive for antigenemia, acute and convalescent sera were available from two children to test for rotavirus-specific antibodies by EIA. Both children had a greater than 4-fold increase in rotavirus-specific antibody titers from acute to convalescent samples (boxes, Figure 1B), indicating that at least two of these five children had rotavirus infections undiagnosed by stool testing.

Children with gastroenteritis were further stratified upon the basis of the presence or absence of the clinical manifestation of diarrhea. A comparable high mean OD (*p* = 0.88) and rate (*p* = 1.00) of antigenemia was observed in serum from children with rotavirus-positive stool and diarrhea (43/48; 90%), and in children with rotavirus-positive stool without diarrhea (8/9; 89%; Figure 1B). For comparison, there was a significant difference in mean OD (*p* = 0.001) and antigenemia rate (*p* = 0.001) in children with rotavirus-positive stools without diarrhea compared to children with noninfectious, nonchronic conditions (Figure 1A and 1B).

### Detection of Antigenemia in Ill Children without Gastroenteritis

The finding that rotavirus antigenemia was present in the absence of diarrhea suggested that rotavirus might be present in other populations of ill children. To test whether children with respiratory symptoms, but no diarrhea, might have rotavirus antigenemia, we tested 17 serum samples from children suffering from bronchiolitis not attributed to any of several respiratory viruses tested (see Methods). Of these serum samples, two were rotavirus positive (12%, data not shown). A total of 58 serum samples tested from children with bronchiolitis attributed to infection with a respiratory virus were negative for rotavirus antigen (data not shown). The rate of rotavirus antigenemia in undiagnosed bronchiolitis was significant compared to the rate in diagnosed bronchiolitis (*p* = 0.049). As many as 66% of children with rotavirus infection exhibit upper respiratory tract symptoms in addition to gastroenteritis [40]. However, it is unknown whether children with rotavirus infection have respiratory disease in the absence of gastroenteritis. Our findings suggest that rotavirus infection may be underdiagnosed and highlight a possible etiologic role for rotavirus in respiratory illness in some children.

### Neither Time after Onset of Gastroenteritis Nor Age Affected Level of Rotavirus Antigenemia Early after Onset of Symptoms

**Figure 2:**
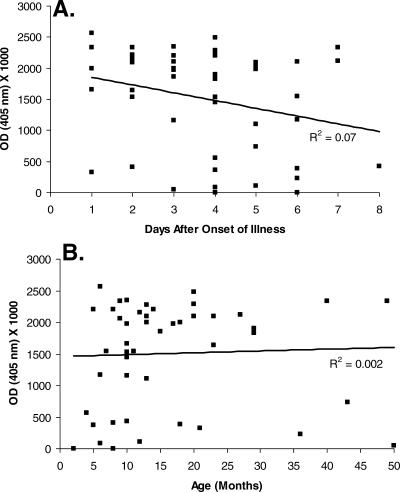
Detection of Rotavirus Antigenemia within Eight Days after Onset of Symptoms Does Not Depend on the Number of Days after Onset of Illness or the Age of Child The ODs of serum samples obtained from children with rotavirus-positive stools were plotted against days after onset of illness (A) or age of child in months (B). Correlation analysis was performed (line) and *R* ^2^ values calculated.

Analysis of small numbers of samples collected from zero to 22 days after onset of diarrhea suggested that time after onset of diarrhea can negatively affect the detection of rotavirus antigenemia or RNA [16,19,20]. However, rotavirus is an acute infection and generally cleared within seven to 14 days. Most of the samples examined in Figure 1 were collected within six days after the onset of disease, and all sera were collected from children sufficiently ill to be hospitalized on the day of serum collection. To investigate whether time within the first week after onset of disease affected antigenemia, we plotted the antigenemia OD values from the rotavirus-positive stool group (*n* = 57) against days after onset of gastroenteritis (Figure 2A). The correlation between serum antigen levels and longer time after onset of gastroenteritis (*n* = 5–11 values/day) was not statistically significant in this patient group (*r* = −0.04, *f* = 0.14, and *p* = 0.09).

Age at onset of illness might adversely affect detection of antigenemia. To determine whether age was associated with antigenemia levels, the antigenemia OD values from the rotavirus-positive stool group were plotted against the age of the child (between 2.5–50 mo) from whom each sample was obtained (Figure 2B). No significant correlation between serum antigenemia level and age of the child was observed (*r* = 0.03, *p* = 0.83)

### The Detection of Rotavirus Antigenemia Negatively Correlates with Acute Antibody Titers

Children with low rotavirus-specific serum IgG titers have been shown to have higher levels of rotavirus antigen in their serum [20], suggesting that serum antibodies influence the ability to detect antigenemia. We tested whether higher levels of acute rotavirus-specific IgA or IgG serum antibodies resulted in a lower antigenemia OD value. There was a significant negative correlation between the OD value from the EIA to detect antigenemia and IgA titers (*n* = 39 samples, *r* = −0.44) (95% confidence interval −0.66 to −0.15; *p* = 0.002) and IgG (*n* = 39 samples, *r* = −0.40) (95% confidence interval −0.63 to −0.11; *p* = 0.007) titers. There was no correlation between convalescent antirotavirus IgG and IgA titers and the presence of rotavirus antigenemia. Comparison of acute rotavirus-specific IgA and IgG levels between rotavirus-negative children with diarrhea to those of the rotavirus-positive children with diarrhea found no statistically significant trend of higher acute antibody levels in the children without rotavirus, (*p* = 0.12 and *p* = 0.19, respectively). However, inclusion of the acute rotavirus-specific antibody titers of the rotavirus-negative children to the correlation analysis still resulted in a clear negative correlation between acute IgA (*n* = 75 samples, *r* = −0.33) (95% confidence interval −0.52 to −0.11) or IgG titer (*n* = 75 samples, *r* = −0.37) (95% confidence interval −0.55 to −0.16) and antigenemia.

There are two possible explanations for the inverse correlation between antigenemia detection and the level of serum antibodies: (1) serum antibody prevents antigenemia or (2) serum antibody is bound to rotavirus antigens present in the blood, preventing detection of the antigens by EIA. Further analysis will be necessary to determine if stool rotavirus-positive children with low or undetectable levels of antigenemia have rotavirus antibody-antigen complexes present in the circulation.

### Rotavirus Strain Does Not Influence Antigenemia

The high rate of antigenemia in children (Figure 1) suggests that antigenemia is not restricted to a specific strain or serotype of rotavirus. To test this hypothesis, the P and G genotypes of the virus in the stool were typed using reverse transcriptase-PCR (Table 1). Several children had mixed infections based on detection of multiple genotypes. Analysis of variance among serum antigenemia values by either G or P type indicated no significant association between type of the virus and antigenemia (*p* = 0.96 and *p* = 0.78, respectively). This lack of association between antigenemia and viral G or P type suggests that antigenemia is not a property of a viral type.

**Table 1:**
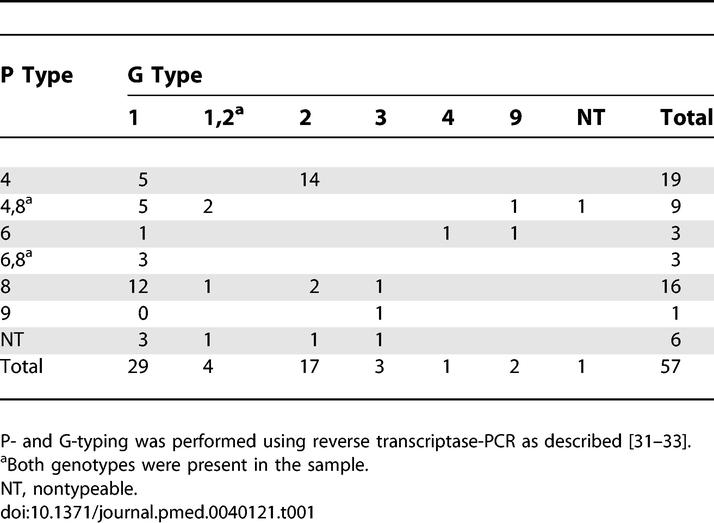
Distribution of P and G Genotypes in Rotavirus-Positive Stools

### Detection of Infectious Virus in Rotavirus-Positive Sera

**Figure 3:**
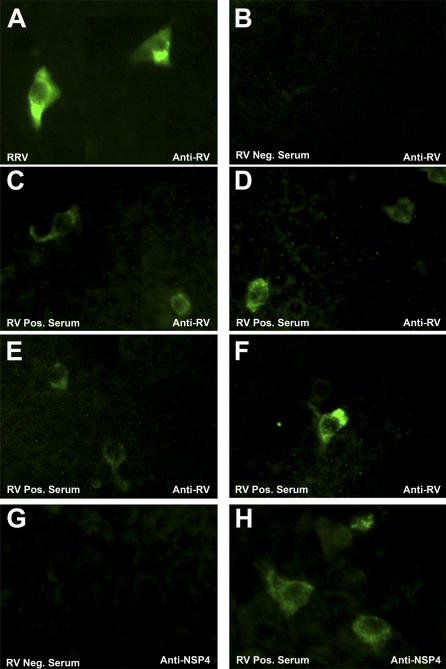
Infectious Rotavirus Is Present in Children with Rotavirus Antigenemia A total of 11 antigen-positive and nine antigen-negative sera were examined for infectious virus by focus-forming assay after three blind serial passes in HT-29 cells. Infected HT-29 cells were identified using a rabbit antiserum against the ALA strain of rotavirus (A–F) or against NSP4 peptide 114–135 (G, H) followed by a FITC-labeled secondary antibody. Shown are: (A) Rhesus rotavirus (RRV) as a positive control; (B, G) Antigen negative serum; and (C–F, H) antigen positive serum.

Reports of the detection of RNA in the blood of children infected with rotavirus [10,16,19,20,22,41] suggest, but do not prove, that intact infectious virions are present. To test whether detection of antigenemia in serum implied that infectious virus was present, serum aliquots were diluted 1:20 to 1:160 and subjected to three to five serial blind passages in human colon adenocarcinoma cells (HT-29), followed by a fluorescent focus assay (Figure 3). Sera were diluted, (1) because of the expectation that the presence of serum inhibitors of viral replication [42,43] would inhibit detection of virus in undiluted or low dilutions of sera, and (2) to provide an indication of virus titer in the original sera. Fluorescent foci indicative of infectious virus were observed at either a 1:80 or 1:160 dilution in 11/11 (100%) sera from serum antigen-positive children and in two out of nine (22%) sera from serum antigen-negative individuals (Table 2). The two children with viremia from the latter group were positive for stool rotavirus. Statistical analysis indicated that antigenemia was predictive of viremia in children (*p* = 0.002, Yates' corrected χ^2^). A subset of sera (Table 2) was analyzed at additional dilutions, 1:320 to 1:2,560, and serially passaged. Although we were unable to determine virus titers directly from the blood, limiting dilution analyses showed infectious virus at a maximal dilution of 1:160.

**Table 2:**
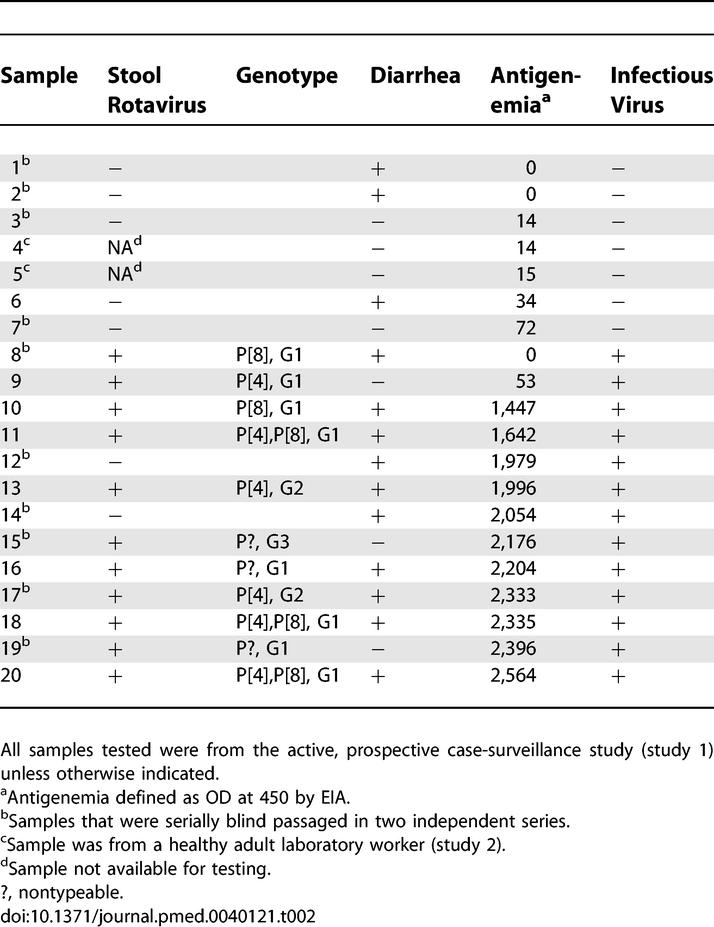
Rotavirus Viremia in Children with Antigenemia

## Discussion

Rotavirus infection in children results in antigenemia [19,20]. Although RNA has been associated with antigenemia [20], proof of viremia in children has not been previously demonstrated. Here, we present proof that antigenemia is associated with infectious virus in rotavirus-infected children. Major technical barriers to the isolation of infectious wild-type human rotavirus from serum are that human primary isolates require adaptation to grow in vitro [34] and that serum factors can inhibit viral replication [42,43]. Attempts to isolate human rotavirus from serum were unsuccessful using MA104 cells [20]. Our detection of infectious virus in serum may have been greatly enhanced by the use of HT-29 cells [34], serial blind passage, and testing multiple dilutions of sera. The detection of infectious virus in the serum from rotavirus-infected children indicates that rotavirus is present systemically, in addition to its well-documented mucosal infection. In addition, antigenemia is predictive of viremia and can be used as a marker for the presence of extraintestinal rotavirus.

The possible clinical consequences of viremia in humans are not yet known. During viremia, infectious rotavirus would gain access to most tissues and cell types in the body. Indeed, rotavirus proteins or RNA have been detected in endothelial cells [14,15], liver [22], central nervous system and cerebrospinal fluid [12,14], and kidney [22] from infected children. The detection of infectious virus in serum suggests that one possible mechanism of how virus reaches these sites involves a viremic dissemination of rotavirus extraintestinally.

Here, we observed an antigenemia rate of ∼90% in samples obtained from children with rotavirus diarrhea, which is substantially higher than previous reports (43% [20], 63% [21], and 67% [19]). The differences in rates observed among the studies may be attributed to differences in duration of illness prior to serum sample collection. In the current study, all children analyzed had gastroenteritis for less than seven days and were sufficiently ill to be hospitalized on the day of serum collection. As one might expect in an acute viral infection, the rates of antigenemia are greater when serum from children with diarrhea less than eight days are included, and as duration of illness increased, the likelihood of antigenemia detection decreased [19,20]. Antigenemia was also less likely to be detected in children with higher serum antibody titers [20]. Therefore, based upon the current results and studies in animal models of rotavirus infection [19,24,38,44], we suspect that close to 100% of children with rotavirus infection exhibit antigenemia early in infection, and the rate declines over time as acute antibody titers or other immune effectors rise. A large prospective study examining sera from children on specific days after the onset of symptoms or repeated serum samplings over time from infected children will be necessary to exactly determine the rate of occurrence of antigenemia in relation to the onset of symptoms and the influence of antibody titer upon detection. These studies will also be necessary to determine whether there is a relationship between the presence of type-specific neutralizing antibodies and the occurrence of antigenemia.

Previously, rotavirus antigenemia was reported in children with rotavirus-positive stools and diarrhea [19–21]. Because previous studies examined antigenemia in children with diarrhea, it was not known if diarrhea was necessary for antigenemia. We found that antigenemia occurred in eight out of nine rotavirus-positive children in the absence of diarrhea (Figure 1B). Because of this finding and the association of rotavirus infection with respiratory disease [40,45–47], we tested whether rotavirus antigenemia was present in children with nongastrointestinal illness by examining children with bronchiolitis for antigenemia. We found two out of 17 serum samples were rotavirus-antigen positive. This finding may be incidental, or alternatively it may support previous associations of rotavirus infection with respiratory disease and indicate that diarrhea may not be the sole clinical manifestation associated with rotavirus antigenemia/viremia. In fact, studies in animal models indicate that viremia occurs in the absence of diarrhea [19,24] and that infectious virus is present within the respiratory tract [23,38,44].

We also observed an antigenemia rate of 12% in children with diarrhea but with rotavirus-negative stools. There are several possible explanations for this finding, including discordance between the kinetics of stool and sera antigenemia, a false-negative stool result, the occurrence of rotavirus disease in the absence of detectable stool antigen excretion (by current assays), or a false positive antigenemia result. The latter is unlikely because rotavirus infection was confirmed by a greater than 4-fold rise in convalescent antibody titer [29,48], as well as the fact that neither seroconversion nor antigenemia was detected in either serum from children with noninfectious, nonchronic conditions or healthy adults. These findings confirm previous suggestions [19,20] that testing serum for rotavirus antigen may be an alternate approach to diagnose rotavirus infection.

Our data suggest that most children infected with rotavirus develop viremia, but it does not appear that systemic infection routinely causes clinically significant nongastrointestinal disease. It is more probable that rotavirus viremia results in subclinical extraintestinal infection that in some cases, may progress to clinical diseases. This hypothesis may explain the broad array of diseases associated with rotavirus infection, including biliary atresia, pneumonia, disseminated intravascular coagulation, and Reye's syndrome. Alternatively, because rotavirus is such a common infection, these associations may have been coincidental. However, the finding that rotavirus commonly causes viremia suggests a re-evaluation of the association of rotavirus with other nonintestinal diseases is warranted. Because the number of children infected with rotavirus each year is over 114 million [1], even a small percentage of these children experiencing serious clinical extraintestinal disease would mean a relatively large number of children could be affected. The discovery that rotavirus causes viremia in children changes the way we view rotavirus pathogenesis, and future work will need to focus upon the impact of rotavirus viremia upon disease burden.

## Abbreviations

EIA: enzyme immunoassay
IgA: Immunoglobulin A
IgG: Immunoglobulin G
OD: optical density

## References

[pmed-0040121-b001] Glass RI, Parashar UD, Bresee JS, Turcios R, Fischer TK (2006). Rotavirus vaccines: Current prospects and future challenges. Lancet.

[pmed-0040121-b002] Tucker AW, Haddix AC, Bresee JS, Holman RC, Parashar UD (1998). Cost-effectiveness analysis of a rotavirus immunization program for the United States. JAMA.

[pmed-0040121-b003] Gilger MA, Matson DO, Conner ME, Rosenblatt HM, Finegold MJ (1992). Extraintestinal rotavirus infections in children with immunodeficiency. J Pediatr.

[pmed-0040121-b004] Santosham M, Yolken RH, Quiroz E, Dillman L, Oro G (1983). Detection of rotavirus in respiratory secretions of children with pneumonia. J Pediatr.

[pmed-0040121-b005] Ruzicka T, Rosendahl C, Braun-Falco O (1985). A probable case of rotavirus exanthem. Arch Dermatol.

[pmed-0040121-b006] Limbos MA, Lieberman JM (1996). Disseminated intravascular coagulation associated with rotavirus gastroenteritis: Report of two cases. Clin Infect Dis.

[pmed-0040121-b007] Takahashi S, Oki J, Miyamoto A, Koyano S, Ito K (1999). Encephalopathy associated with haemophagocytic lymphohistiocytosis following rotavirus infection. Eur J Pediatr.

[pmed-0040121-b008] Yoshida A, Kawamitu T, Tanaka R, Okumura M, Yamakura S (1995). Rotavirus encephalitis: Detection of the virus genomic RNA in the cerebrospinal fluid of a child. Pediatr Infect Dis J.

[pmed-0040121-b009] Nigrovic LE, Lumeng C, Landrigan C, Chiang VW (2002). Rotavirus cerebellitis?. Clin Infect Dis.

[pmed-0040121-b010] Nishimura S, Ushijima H, Nishimura S, Shiraishi H, Kanazawa C (1993). Detection of rotavirus in cerebrospinal fluid and blood of patients with convulsions and gastroenteritis by means of the reverse transcription polymerase chain reaction. Brain Dev.

[pmed-0040121-b011] Pang XL, Joensuu J, Vesikari T (1996). Detection of rotavirus RNA in cerebrospinal fluid in a case of rotavirus gastroenteritis with febrile seizures. Pediatr Infect Dis J.

[pmed-0040121-b012] Lynch M, Shieh WJ, Tatti K, Gentsch JR, Ferebee-Harris T (2003). The pathology of rotavirus-associated deaths, using new molecular diagnostics. Clin Infect Dis.

[pmed-0040121-b013] Iturriza-Gomara M, Auchterlonie IA, Zaw W, Molyneaux P, Desselberger U (2002). Rotavirus gastroenteritis and central nervous system (CNS) infection: Characterization of the VP7 and VP4 genes of rotavirus strains isolated from paired fecal and cerebrospinal fluid samples from a child with CNS disease. J Clin Microbiol.

[pmed-0040121-b014] Morrison C, Gilson T, Nuovo GJ (2001). Histologic distribution of fatal rotaviral infection: An immunohistochemical and reverse transcriptase in situ polymerase chain reaction analysis. Hum Pathol.

[pmed-0040121-b015] Cioc AM, Nuovo GJ (2002). Histologic and in situ viral findings in the myocardium in cases of sudden, unexpected death. Mod Pathol.

[pmed-0040121-b016] Chiappini E, Azzari C, Moriondo M, Galli L, de Martino M (2005). Viraemia is a common finding in immunocompetent children with rotavirus infection. J Med Virol.

[pmed-0040121-b017] Nuovo GJ, Owor G, Andrew T, Magro C (2002). Histologic distribution of fatal rotaviral pneumonitis: an immunohistochemical and RT in situ PCR analysis. Diagn Mol Pathol.

[pmed-0040121-b018] Saulsbury FT, Winkelstein JA, Yolken RH (1980). Chronic rotavirus infection in immunodeficiency. J Pediatr.

[pmed-0040121-b019] Blutt SE, Kirkwood CD, Parreno V, Warfield KL, Ciarlet M (2003). Rotavirus antigenaemia and viraemia: A common event?. Lancet.

[pmed-0040121-b020] Fischer TK, Ashley D, Kerin T, Reynolds-Hedmann E, Gentsch J (2005). Rotavirus antigenemia in patients with acute gastroenteritis. J Infect Dis.

[pmed-0040121-b021] Nakagomi T, Nakagomi O (2005). Rotavirus antigenemia in children with encephalopathy accompanied by rotavirus gastroenteritis. Arch Virol.

[pmed-0040121-b022] Li N, Wang ZY (2003). Viremia and extraintestinal infections in infants with rotavirus diarrhea. Di Yi Jun Yi Da Xue Xue Bao.

[pmed-0040121-b023] Fenaux M, Cuadras MA, Feng N, Jaimes M, Greenberg HB (2006). Extraintestinal spread and replication of a homologous EC rotavirus strain and a heterologous rhesus rotavirus in BALB/c mice. J Virol.

[pmed-0040121-b024] Blutt SE, Fenaux M, Warfield KL, Greenberg HB, Conner ME (2006). Active viremia in rotavirus-infected mice. J Virol.

[pmed-0040121-b025] Blutt SE, Conner ME (2007). Rotavirus: To the gut and beyond!. Curr Opin Gastroenterol.

[pmed-0040121-b026] Lee BP, Azimi PH, Staat MA, Louie L, Parada E (2005). Nonmedical costs associated with rotavirus disease requiring hospitalization. Pediatr Infect Dis J.

[pmed-0040121-b027] Staat MA, Azimi PH, Berke T, Roberts N, Bernstein DI (2002). Clinical presentations of rotavirus infection among hospitalized children. Pediatr Infect Dis J.

[pmed-0040121-b028] O'Neal CM, Crawford SE, Estes MK, Conner ME (1997). Rotavirus VLPs administered mucosally induce protective immunity. J Virol.

[pmed-0040121-b029] Velazquez FR, Matson DO, Calva JJ, Guerrero L, Morrow AL (1996). Rotavirus infections in infants as protection against subsequent infections. N Engl J Med.

[pmed-0040121-b030] Velazquez FR, Matson DO, Guerrero ML, Shults J, Calva JJ (2000). Serum antibody as a marker of protection against natural rotavirus infection and disease. J Infect Dis.

[pmed-0040121-b031] Gouvea V, Glass RI, Woods P, Taniguchi K, Clark HF (1990). Polymerase chain reaction amplification and typing of rotavirus nucleic acid from stool specimens. J Clin Microbiol.

[pmed-0040121-b032] Gentsch JR, Glass RI, Woods P, Gouvea V, Gorziglia M (1992). Identification of group A rotavirus gene 4 types by polymerase chain reaction. J Clin Microbiol.

[pmed-0040121-b033] Bok K, Matson DO, Gomez JA (2002). Genetic variation of capsid protein VP7 in genotype g4 human rotavirus strains: Simultaneous emergence and spread of different lineages in Argentina. J Clin Microbiol.

[pmed-0040121-b034] Superti F, Tinari A, Baldassarri L, Donelli G (1991). HT-29 cells: A new substrate for rotavirus growth. Arch Virol.

[pmed-0040121-b035] Crawford SE, Mukherjee SK, Estes MK, Lawton JA, Shaw AL (2001). Trypsin cleavage stabilizes the rotavirus VP4 spike. J Virol.

[pmed-0040121-b036] Ball JM, Tian P, Zeng CQ, Morris AP, Estes MK (1996). Age-dependent diarrhea induced by a rotaviral nonstructural glycoprotein. Science.

[pmed-0040121-b037] Ciarlet M, Estes MK (1999). Human and most animal rotavirus strains do not require the presence of sialic acid on the cell surface for efficient infectivity. J Gen Virol.

[pmed-0040121-b038] Crawford SE, Patel DG, Cheng E, Berkova Z, Hyser JM (2006). Rotavirus viremia and extraintestinal viral infection in the neonatal rat model. J Virol.

[pmed-0040121-b039] Dean AG, Dean JA, Burton AH, Dicker RC (1991). Epi Info: A general-purpose microcomputer program for public health information systems. Am J Prev Med.

[pmed-0040121-b040] Lewis HM, Parry JV, Davies HA, Parry RP, Mott A (1979). A year's experience of the rotavirus syndrome and its association with respiratory illness. Arch Dis Child.

[pmed-0040121-b041] Ushijima H, Xin KQ, Nishimura S, Morikawa S, Abe T (1994). Detection and sequencing of rotavirus VP7 gene from human materials (stools, sera, cerebrospinal fluids, and throat swabs) by reverse transcription and PCR. J Clin Microbiol.

[pmed-0040121-b042] Beisner B, Kool D, Marich A, Holmes IH (1998). Characterisation of G serotype dependent non-antibody inhibitors of rotavirus in normal mouse serum. Arch Virol.

[pmed-0040121-b043] Smith EM, Estes MK, Graham DY, Gerba CP (1979). A plaque assay for the simian rotavirus SAII. J Gen Virol.

[pmed-0040121-b044] Azevedo MS, Yuan L, Jeong KI, Gonzalez A, Nguyen TV (2005). Viremia and nasal and rectal shedding of rotavirus in gnotobiotic pigs inoculated with Wa human rotavirus. J Virol.

[pmed-0040121-b045] Carr ME, McKendrick GD, Spyridakis T (1976). The clinical features of infantile gastroenteritis due to rotavirus. Scand J Infect Dis.

[pmed-0040121-b046] Zhaori GT, Fu LT, Xu YH, Guo YR, Peng ZJ (1991). Detection of rotavirus antigen in tracheal aspirates of infants and children with pneumonia. Chin Med J (Engl).

[pmed-0040121-b047] Uhnoo I, Svensson L (1986). Clinical and epidemiological features of acute infantile gastroenteritis associated with human rotavirus subgroups 1 and 2. J Clin Microbiol.

[pmed-0040121-b048] Rodriguez WJ, Kim HW, Arrobio JO, Brandt CD, Chanock RM (1977). Clinical features of acute gastroenteritis associated with human reovirus-like agent in infants and young children. J Pediatr.

